# Myeloid-resident neuropilin-1 influences brown adipose tissue in obesity

**DOI:** 10.1038/s41598-021-95064-w

**Published:** 2021-08-03

**Authors:** Roberto Diaz-Marin, Sergio Crespo-Garcia, Ariel M. Wilson, Manuel Buscarlet, Agnieszka Dejda, Frédérik Fournier, Rachel Juneau, Thierry Alquier, Przemyslaw Sapieha

**Affiliations:** 1 Department of Biochemistry, Maisonneuve-Rosemont Hospital Research Centre, Université de Montréal, 5415 Assumption Boulevard, Montréal, QC H1T 2M4 Canada; 2 Department of Ophthalmology, Maisonneuve-Rosemont Research Centre, Université de Montréal, Montréal, QC H1T2M4 Canada; 3 Montreal Diabetes Research Centre and Centre de Recherche du Centre Hospitalier de l’Université de Montréal (CRCHUM), 900 rue Saint-Denis, Montréal, QC H2X0A9 Canada

**Keywords:** Cell biology, Immunology, Diseases

## Abstract

The beneficial effects of brown adipose tissue (BAT) on obesity and associated metabolic diseases are mediated through its capacity to dissipate energy as heat. While immune cells, such as tissue-resident macrophages, are known to influence adipose tissue homeostasis, relatively little is known about their contribution to BAT function. Here we report that neuropilin-1 (NRP1), a multiligand single-pass transmembrane receptor, is highly expressed in BAT-resident macrophages. During diet-induced obesity (DIO), myeloid-resident NRP1 influences interscapular BAT mass, and consequently vascular morphology, innervation density and ultimately core body temperature during cold exposure. Thus, NRP1-expressing myeloid cells contribute to the BAT homeostasis and potentially its thermogenic function in DIO.

## Introduction

Obesity has reached pandemic proportions in the Western world and represents a major risk factor for the development of type 2 diabetes, cardiovascular diseases and cancer^1,2^. It results from an imbalance between energy intake and energy expenditure causing an increment of fat mass in adipose tissue or liver. Adipose tissue can be classified as white adipose tissue (WAT), brown adipose tissue (BAT) or beige adipose tissue (BgAT)^3–6^. WAT participates mainly in energy storage and release^3^ whereas BAT and BgAT can metabolize stored lipids to produce heat by a process known as nonshivering thermogenesis or adaptive thermogenesis^4,5^. This type of thermogenesis occurs in the mitochondria and is mediated by uncoupling protein 1 (UCP1), which facilitates the uncoupling of electrons from the synthesis of adenosine triphosphate (ATP) towards heat production^4^. Obesity is correlated with an increase in WAT mass and impaired BAT activity^7,8^. It has been proposed that potentiating thermogenesis in BAT could influence weight gain in humans^9,10^.

Adipose tissues are rich in effectors of both innate and adaptive immunity^11,12^ whose numbers are altered with obesity^13,14^. The contribution of the innate immune system and specifically adipose tissue macrophages to low-grade inflammation and homeostasis in adipose tissue has been well documented^15–17^. Macrophage accretion in adipose tissue can increase inflammatory cytokines and lead to chronic low-grade inflammation^17–19^. With regards to BAT, alternatively activated macrophages have been suggested to influence BAT homeostasis and thermogenesis^20^. However, these findings were recently challenged^21^ raising questions about the involvement of macrophages in BAT activation.

While investigating the role of innate immunity in obesity, we previously identified myeloid-resident neuropilin-1 (NRP1) as necessary for healthy weight gain and maintaining glucose tolerance in obesity^22^. NRP1 is a single pass transmembrane receptor that binds multiple ligands (e.g. semaphorins, VEGFs) and receptors (e.g. VEGFR2, integrins, plexins), influences intracellular signaling^23^, axonal guidance^24^ and neuronal and vascular development^25^. Adipose tissue macrophages (ATMs) devoid of NRP1 are less efficient at internalizing lipids and shift their metabolism towards a more pro-inflammatory carbohydrate-based glycolytic metabolism^22^. Here we investigated the role of NRP1 expressing myeloid cells on BAT biology and the control of thermogenesis.

## Results

### Interscapular brown adipose tissue macrophages express high levels of neuropilin-1

**Figure 1:**
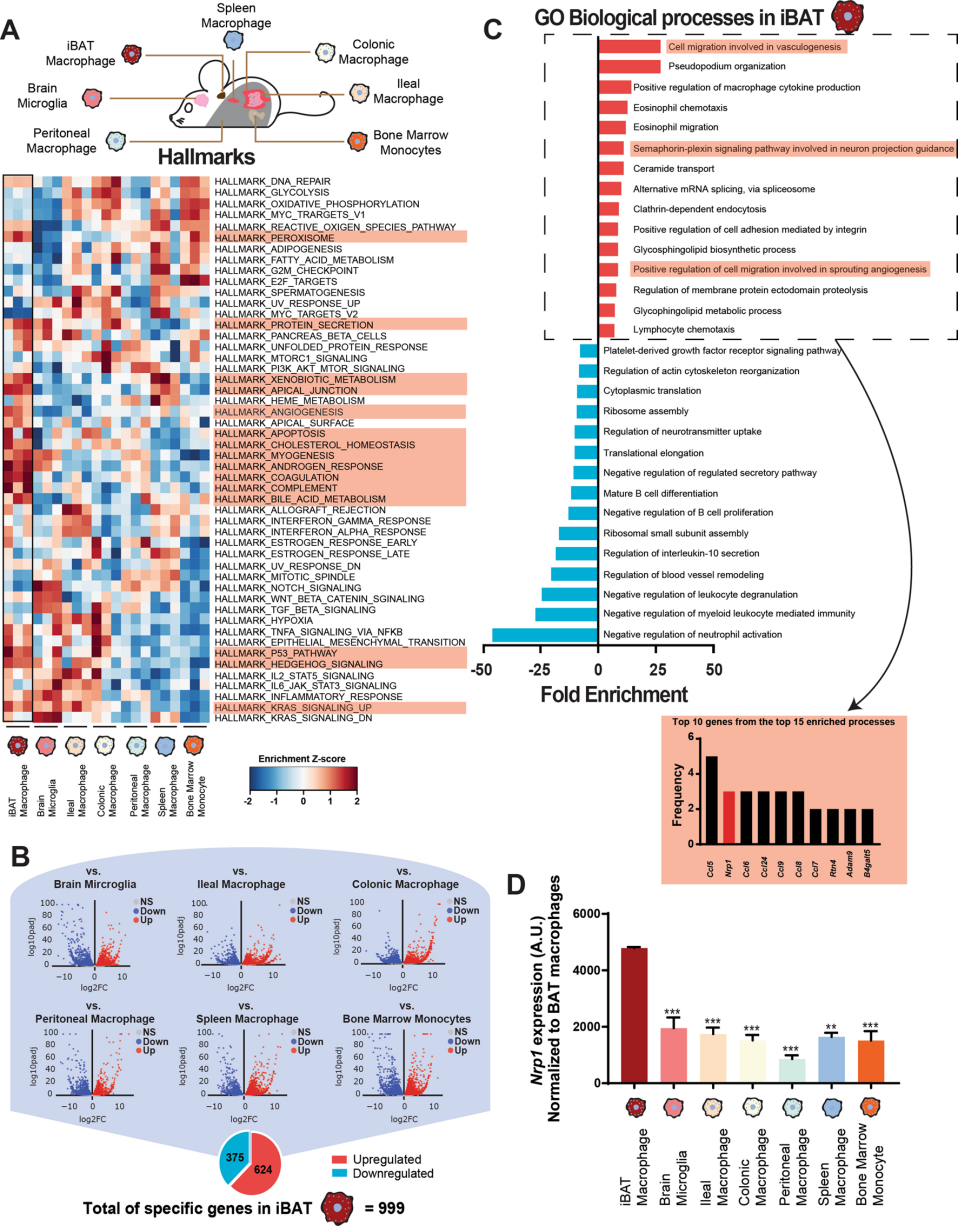
Interscapular brown adipose tissue macrophages express high levels of neuropilin-1. (**A**) Heat map showing differentially regulated genes in various tissue-resident macrophages compared to iBAT macrophages, from RNA-seq analysis (n = 3 per group). (**B**) Representation of significantly upregulated or down regulated genes in iBAT-specific macrophages. (**C**) Gene ontology (GO) categories of the top 15 upregulated and downregulated biological processes associated with iBAT-specific macrophages. (**D**) *Nrp1* expression in macrophages isolated from distinct tissues of *C57BL/6* mice normalized to the expression levels of BAT macrophages (n = 3).

In order to investigate the transcriptomic signatures of interscapular brown adipose tissue (iBAT) macrophages compared to resident macrophage populations of other tissues, we analyzed the transcriptomes of fluorescence-activated cell sorted (FACS) native macrophages across various organs^26^. We ran Gene Set Variation Analysis (GSVA) to identify enriched gene sets in iBAT-resident macrophages (iBAT-MPs) compared to macrophages from other tissues. We found enrichment in distinct gene sets across all macrophage populations (Fig. 1A, Supplemental Table S1). Six differential gene expression analyses by DESeq2 were performed between iBAT-MPs and all the other tissue macrophage populations; we identified 624 significantly upregulated and 375 significantly downregulated genes in a minimum of 5 out of 6 comparisons that were associated with iBAT-MPs (Fig. 1B, Supplemental Table S2–S3). Interestingly, by analyzing Gene Ontology (GO) biological processes, we found transcript enrichment in iBAT-MPs for cell migration involved in vasculogenesis, semaphorin-plexin signalling pathways and positive regulation of cell migration involved in sprouting angiogenesis (Fig. 1C, Supplemental Table S4). In order to identify specific genes of interest associated with the iBAT-MP function, we determined the most recurrent genes within the top 15 Gene Ontology (GO) biological processes. Of interest, the second-most frequently associated gene with the iBAT-MP enrichment signature was neuropilin-1 *(Nrp1*) (Fig. 1C-inset, Supplemental Table S5), a gene which we had previously demonstrated to play a critical role in white adipose tissue macrophage function^22^. Therefore, we assessed *Nrp1* expression across screened macrophages and found iBAT-MP to express the highest levels of *Nrp1* (Fig. 1D). These data raise the possibility that NRP1 may play a role in the function of iBAT-resident macrophages.

### Neuropilin-1-expressing myeloid cells influence interscapular brown adipose tissue composition in diet-induced obesity

**Figure 2:**
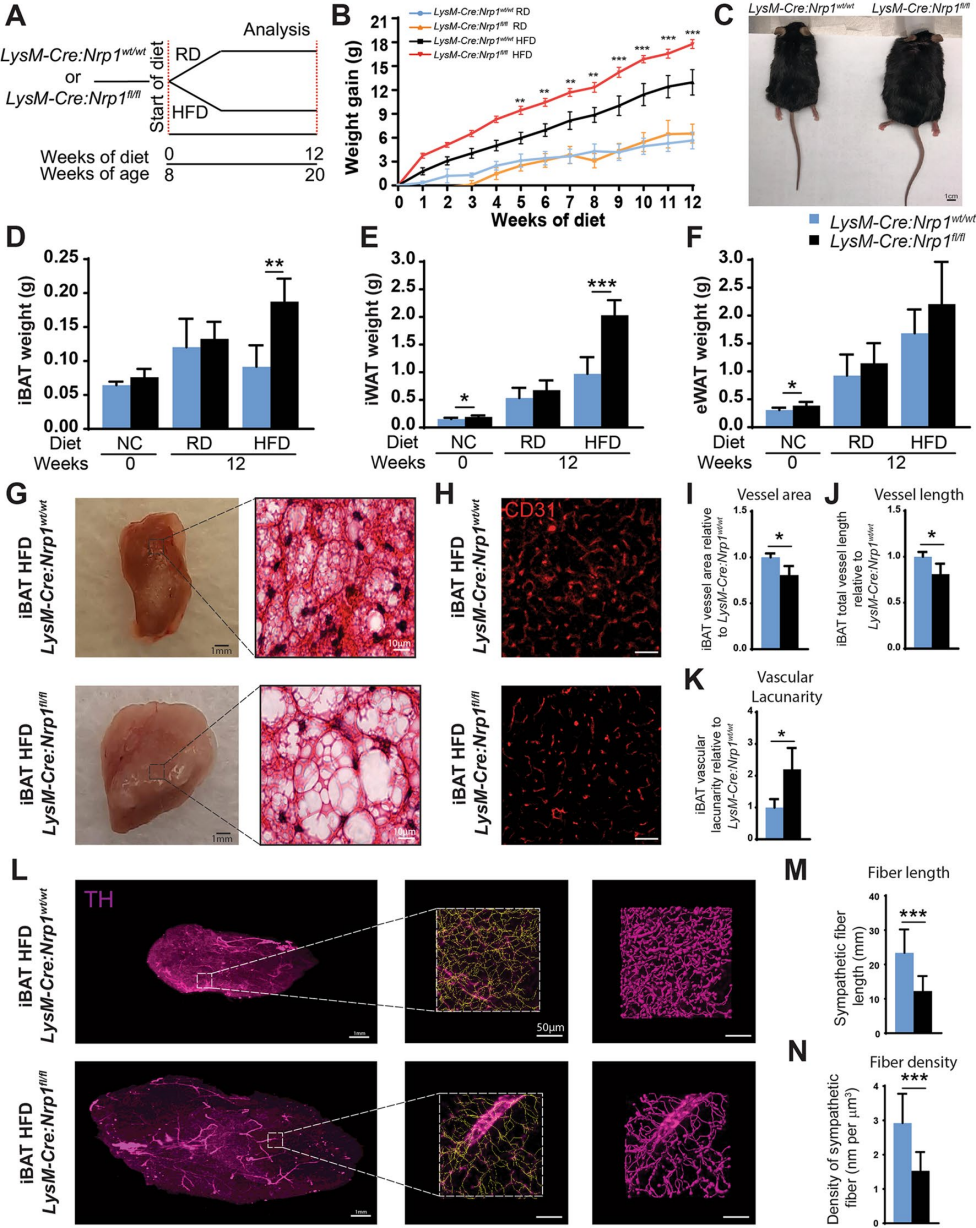
Neuropilin-1-expressing myeloid cells influence interscapular brown adipose tissue composition in diet-induced obesity. (**A**) Schematic representation of the experimental design. At 8 weeks of age, mice were fed with a high-fat diet (HFD) or a matched regular diet (RD) up to 12 weeks. (**B**) Weight gain of *LysM-Cre:Nrp1*^*fl/fl*^, *LysM-Cre:Nrp1*^*wt/wt*^ mice on RD or HFD for 12 weeks (n = 4–9). (**C**) Representative photographs of *LysM-Cre:Nrp1*^*wt/wt*^ and *LysM-Cre:Nrp1*^*fl/fl*^ mice undergoing HFD. (**D**) iBAT, (**E**) iWAT and (**F**) eWAT masses at different time-points after RD or HFD diet feeding (n = 4–6). (**G**) Representative images of fat pads and their corresponding H&E staining of iBAT from *LysM-Cre:Nrp1*^*wt/wt*^ and *LysM-Cre:Nrp1*^*fl/fl*^ mice fed a high fat diet after 12 weeks of diet, n = 4 mice per genotype. (**H**) Representative images of iBAT from *LysM-Cre:Nrp1*^*wt/wt*^ and *LysM-Cre:Nrp1*^*fl/fl*^ mice stained with CD31, imaged on confocal microscope at 10× magnification; scale bar 50 µm. (**I**–**K**) Quantification of vessel area (**I**) length (**J**) and lacunarity (**K**) per section normalized to *LysM-Cre:Nrp1*^*wt/wt*^ (n = 5 per group). (**L**) Representative top view of 3D reconstructions of iBAT from *LysM-Cre:Nrp1*^*wt/wt*^ and *LysM-Cre:Nrp1*^*fl/fl*^ mice stained with TH, imaged on light sheet microscope at 4 × magnification, using the automatic filament tracing tool in Imaris; TH signal is shown in magenta and reconstructed fibers in yellow. (**M**–**N**) Density of sympathetic fiber length (**M**) and density of sympathetic fiber (**N**) from 12-week HFD-fed *LysM-Cre:Nrp1*^*wt/wt*^ and *LysM-Cre:Nrp1*^*fl/fl*^ mice housed at room temperature. Data are presented as mean ± SEM. *p < 0.05, **p < 0.01; ***p < 0.001.

BAT mass and activity are reported to significantly decrease with obesity, age and in diabetic patients^27–29^. Given that loss of BAT function can impact the accumulation of body fat, we investigated the effect of a myeloid-specific knockdown of *Nrp1* in an experimental model of obesity. Eight-week-old *LysM-Cre:Nrp1*^*fl/fl*^ mice or *LysM-Cre:Nrp1*^*wt/wt*^ control littermates were fed either a high-fat diet (HFD; 60% fat calories) or a matched regular diet (RD; 10% fat calories) for 12 weeks (Fig. 2A). Consistent with our previous work^22^, HFD-fed *LysM-Cre:Nrp1*^*fl/fl*^ mice gained significantly more weight when compared to control *LysM-Cre:Nrp1*^*wt/wt*^ mice (Fig. 2B, C) despite not increasing food intake (Supplemental Figure 1A–B). Following diet-induced obesity, we analyzed distinct adipose tissues: (1) eWAT, which consists of a bilateral intra-abdominal visceral depot attached to the epididymis, (2) interscapular brown adipose tissue (iBAT), which is localized between the scapulae and (3) subcutaneous adipose tissue (iWAT), located between the skin and the muscle fascia anterior to the lower segment of the hind limbs (Supplemental Figure 1C). To determine the degree of adiposity, we measured the total weight of eWAT, iBAT and iWAT fat pads (Fig. 2D–F), and in order to determine if weight gain has an effect on adiposity, we normalized these values to whole-body weight (Supplemental Fig. 1D–G). At the beginning of the diet paradigms (0 weeks), *LysM-Cre:Nrp1*^*fl/fl*^ and control mice showed similar weights of total iBAT (Fig. 2D) while a slight increase in baseline eWAT and iWAT weight was noted in *LysM-Cre:Nrp1*^*fl/fl*^ mice (Fig. 2E,F). When the data were normalized to whole-body weight, only eWAT weight was increased (Supplemental Figure 1D–F). Surprisingly, after 12 weeks of diet, *LysM-Cre:Nrp1*^*fl/fl*^ mice on HFD showed significantly higher total or normalized weights of both iBAT and iWAT when compared to *LysM-Cre:Nrp1*^*wt/wt*^ mice, whereas eWAT showed a trend, but was not significant, despite eWAT weight being significantly higher after 4 weeks of diet in *LysM-Cre:Nrp1*^*fl/fl*^ mice (Fig. 2D–F, Supplemental Figure 1D–M). Fat pad weights did not vary between genotypes on RD at this later time point (Fig. 2D–F). Consistent with this, histological assessment of iBAT with H&E staining revealed that HFD-fed *LysM-Cre:Nrp1*^*fl/fl*^ mice showed signs of hypertrophy in iBAT when compared to *LysM-Cre:Nrp1*^*wt/wt*^ mice (Fig. 2G).

Adipose tissue vasculature plays an essential role in nutrient and oxygen supply to iBAT as well as in heat dissipation^30–32^. We therefore assessed iBAT vascularization by CD31 immunofluorescence staining on sections of iBAT from HFD-fed mice. We observed a decrease in vascularized area per analyzed section (Fig. 2H–J). Importantly, when we accounted for iBAT hypertrophy (iBAT weight), we observed a significant increase in total vessel area and total vessel length in iBAT from *LysM-Cre:Nrp1*^*fl/fl*^ mice as would be expected from an increased organ size (Supplemental Figure 1N–O). We also assessed blood vessel lacunarity, a morphological measure pertaining to gaps and heterogeneity, and detected a significant increase in vascular lacunarity per section, in iBAT from *LysM-Cre:Nrp1*^*fl/fl*^ mice (Fig. 2K). Brown adipose tissue is also highly innervated by sympathetic nerve fibers, which regulate thermogenesis, and changes in innervation can influence lipid storage in iBAT^26,33–35^. We therefore used the iDISCO method adapted for adipose tissue^36^ to clear iBAT from HFD-fed mice and stained for tyrosine hydroxylase (TH) to visualize sympathetic axons and evaluate the impact of myeloid specific *Nrp1*-knockdown on sympathetic innervation. 3D reconstructions of 4–5 randomly selected regions in iBAT were performed to evaluate sympathetic fiber length (Fig. 2L). While we detected a decrease in fiber length in *LysM-Cre:Nrp1*^*fl/fl*^ mice (Fig. 2M), when we normalized fiber length to total iBAT weight to account for the effect of iBAT expansion on innervation, we observed a significant increase in the amount of fibers as expected for a larger tissue (Supplemental Figure 1P). We also evaluated fiber density and we detected a significant decreased fiber density in *LysM-Cre:Nrp1*^*fl/fl*^ mice (Fig. 2N), due to adipocyte hypertrophy. Therefore, the observed decrease in vascularization and sympathetic innervation length in iBAT of *LysM-Cre:Nrp1*^*fl/fl*^ mice is not attributed to a decrease in total vasculature or innervation, but rather to tissue expansion, given the increased size of the adipocytes. These data indicate that upon HFD, NRP1-expressing myeloid cells influence iBAT mass by inducing hypertrophy of the tissue, resulting in an expansion of vascular networks and sympathetic innervation likely due to tissue stretch. Compared to controls, the resulting iBAT in *LysM-Cre:Nrp1*^*fl/fl*^ mice exhibit irregular vascular morphology and innervation of reduced density.

### *LysM-Cre:Nrp1*^*fl/fl*^ mice have decreased core temperature during cold exposure

**Figure 3:**
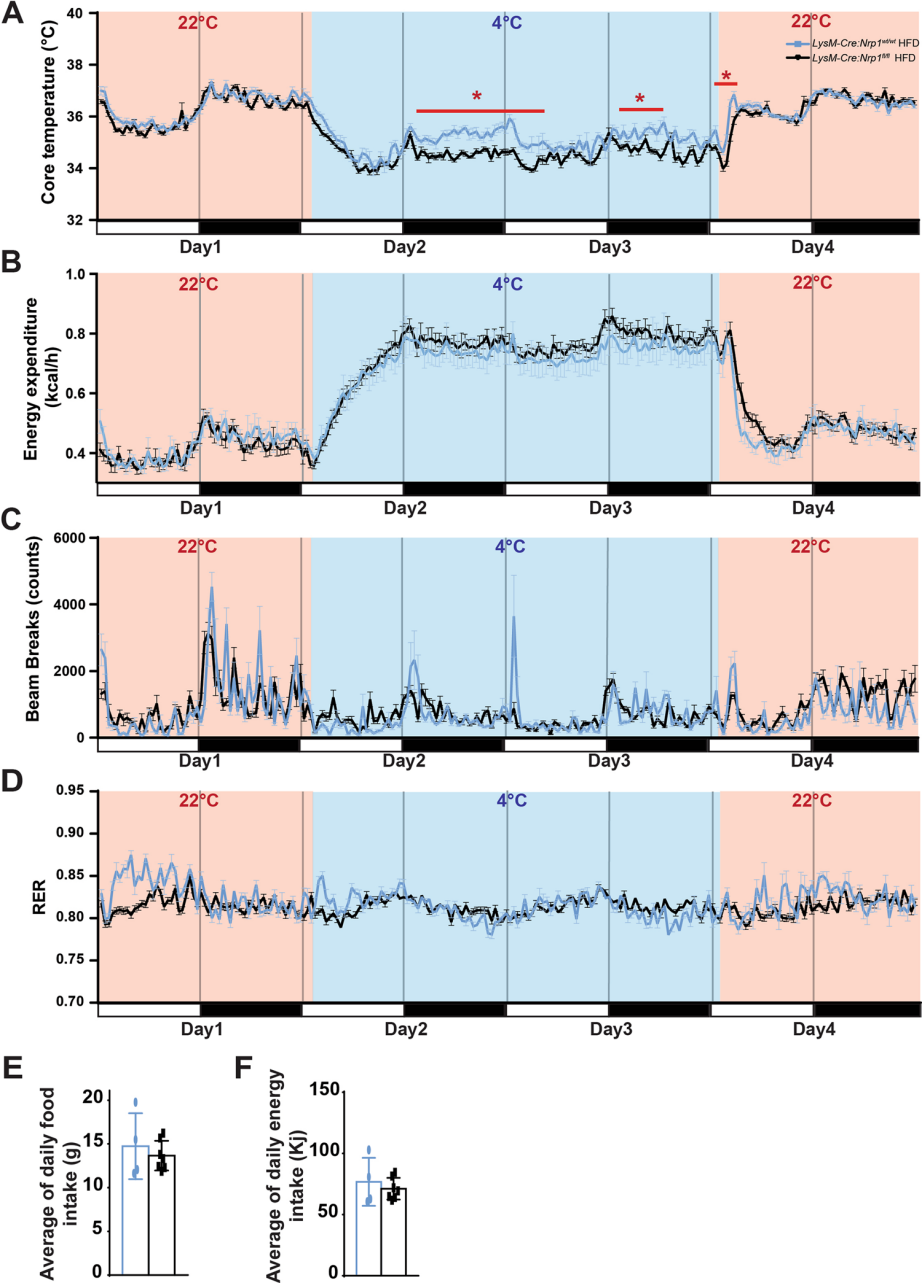
*LysM-Cre:Nrp1*^*fl/fl*^ mice have a decreased core temperature during cold exposure. (**A**) Core temperature, (**B**) Energy expenditure, (**C**) Beam breaks and (**D**) RER of *LysM-Cre:Nrp1*^*wt/wt*^ and *LysM-Cre:Nrp1*^*fl/fl*^ mice fed a high fat diet after 12 weeks of diet, n = 4 mice for *LysM-Cre:Nrp1*^*wt/wt*^ and n = 7 for *LysM-Cre:Nrp1*^*fl/fl*^. (**E**) Average of daily food intake and (**F**) Average of daily energy intake of *LysM-Cre:Nrp1*^*wt/wt*^ and *LysM-Cre:Nrp1*^*fl/fl*^ mice fed a high fat diet after 10–12 weeks of diet, n = 4–7 mice per genotype. Data are presented as mean ± SEM. *p < 0.05; **p < 0.01.

Given the importance of blood vessel perfusion and sympathetic innervation in iBAT-mediated thermogenesis and energy balance, we questioned if the morphological changes observed in iBAT of *LysM-Cre:Nrp1*^*fl/fl*^ mice influenced heat production or energy expenditure. We evaluated this in HFD-fed *LysM-Cre:Nrp1*^*fl/fl*^ or *LysM-Cre:Nrp1*^*wt/wt*^ mice at 12 weeks of diet housed at room temperature for 24 h, followed by cold exposure (4°C) for 48 h then returned to room temperature for 24 h. After an initial 24 h at room temperature, *LysM-Cre:Nrp1*^*fl/fl*^ mice did not show any difference in core temperature when compared to *LysM-Cre:Nrp1*^*wt/wt*^ mice (Fig. 3A). After 24 h of cold exposure, core temperature decreased significantly more in *LysM-Cre:Nrp1*^*fl/fl*^ mice when compared to *LysM-Cre:Nrp1*^*wt/wt*^ (Fig. 3A). Upon return to room temperature, the core temperature of *LysM-Cre:Nrp1*^*fl/fl*^ mice took slightly longer to normalize relative to controls (Fig. 3A). These data suggest that *LysM-Cre:Nrp1*^*fl/fl*^ mice do not adapt to cold as well as control *LysM-Cre:Nrp1*^*wt/wt*^ do. Analysis of energy expenditure between HFD-fed *LysM-Cre:Nrp1*^*fl/fl*^ and *LysM-Cre:Nrp1*^*wt/wt*^ mice did not reveal significant differences between groups (Fig. 3B, Supplemental Figure 2A–C) nor did we observe discrepancies in lower locomotor activity (beam breaks) (Fig. 3C). Since *LysM-Cre:Nrp1*^*fl/fl*^ and *LysM-Cre:Nrp1*^*wt/wt*^ mice had a lower core temperature, yet similar food intake and levels of activity, we next sought to investigate consumption of lipids to fuel systemic metabolism. We thus measured respiratory exchange ratio (RER) during cold exposure by indirect calorimetry to determine the respiratory quotient (ratio between the volume of CO_2_ produced and the volume of O_2_ consumed) that indicates the substrate (e.g. lipid-derived carbohydrate) that is metabolized to supply the body with energy^37^. Consistent with our previous work^22^, RER did not vary between HFD-fed *LysM-Cre:Nrp1*^*fl/fl*^ and *LysM-Cre:Nrp1*^*wt/wt*^ mice both at room temperature and during cold exposure (Fig. 3D). We found that during HFD, lipids become the predominant source of energy or substrate in both control and *LysM-Cre:Nrp1*^*fl/fl*^ mice. Therefore, it is unlikely that the differences observed in core temperature during cold exposure are due to discrepancies in reduced lipid utilization between strains. Furthermore, we evaluated body composition and consistent with our previous work^22^, analysis of animals by Echo MRI showed an increase in fat mass and in percentage of total body fat mass in *LysM-Cre:Nrp1*^*fl/fl*^ while lean mass did not vary (Supplemental Figure 2D–E). The discrepancy in fat mass was not attributed to difference in eating patterns as average daily or total food intake and energy intake from food of *LysM-Cre:Nrp1*^*fl/fl*^ or *LysM-Cre:Nrp1*^*wt/wt*^ mice were similar after 10–12 weeks of HFD whilst housed at room temperature (Fig. 3E,F, Supplemental Figure 1A–B). Altogether, our data suggest that NRP1-expressing myeloid cells impact thermogenic capacity following cold exposure.

### Nonshivering thermogenic stimulation reverses iBAT hypertrophy in *LysM-Cre:Nrp1*^*fl/fl*^ mice

**Figure 4:**
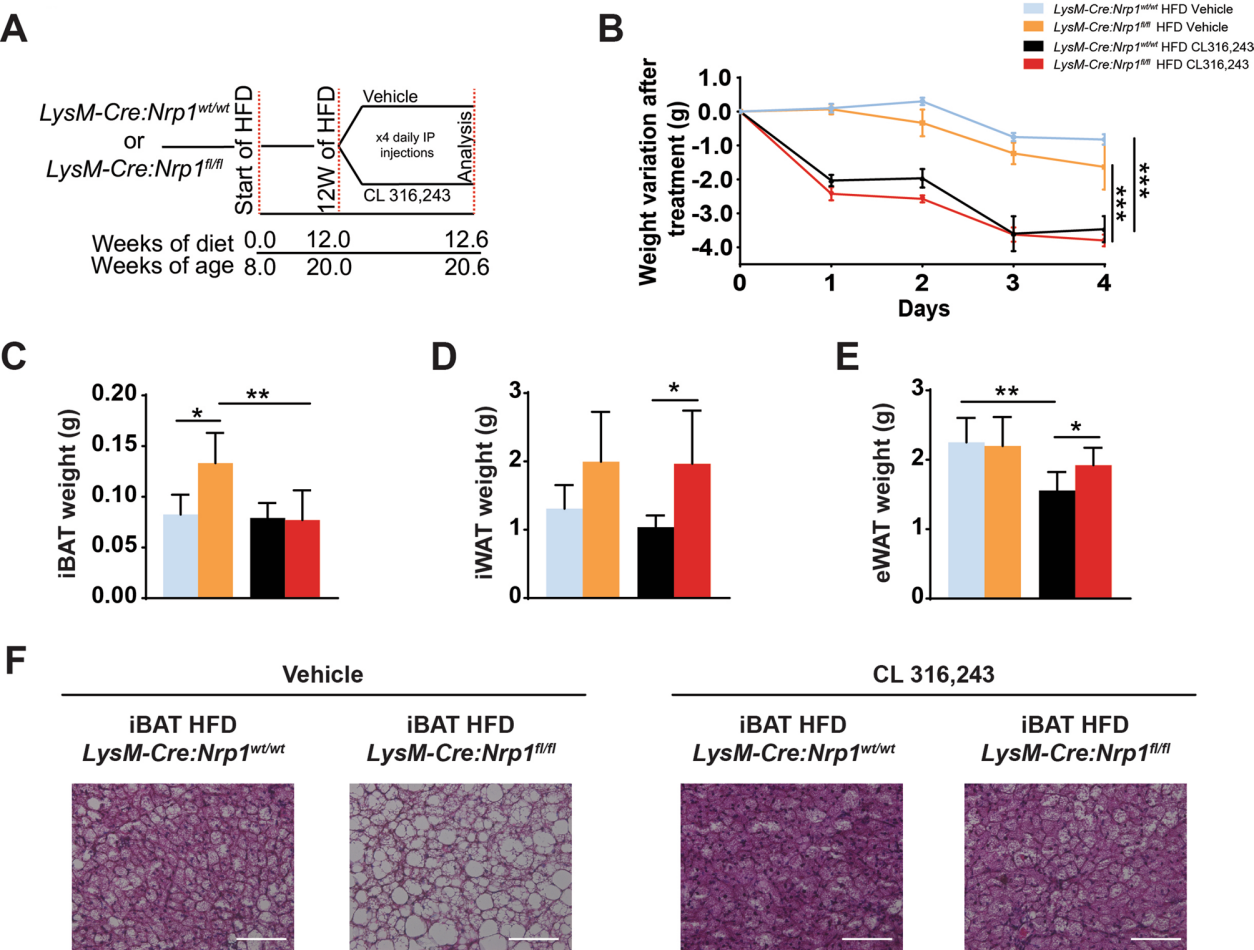
Nonshivering thermogenic stimulation reverses iBAT hyperthrophy in *LysM-Cre:Nrp1*^*fl/fl*^ mice. (**A**) Schematic representation of the experimental design. At 8 weeks of age, mice were fed with a high-fat diet (HFD) up to 12 weeks. At 12 weeks of diet, mice were injected i.p. once daily with CL316,243 or vehicle during 4 consecutive days. (**B**) Weight variation of *LysM-Cre:Nrp1*^*fl/fl*^ and *LysM-Cre:Nrp1*^*wt/wt*^ mice treated with CL316,243 or vehicle (n = 3–4). (**C**) iBAT, (**D**) iWAT and (**E**) eWAT masses after CL316,243 or vehicle treatment (n = 5–7). (**F**) Representative H&E staining of iBAT from *LysM-Cre:Nrp1*^*wt/wt*^ and *LysM-Cre:Nrp1*^*fl/fl*^ mice treated with CL316,243 or vehicle, n = 4 mice per genotype. Data are presented as mean ± SEM. *p < 0.05; **p < 0.01; ***p < 0.001.

Acute cold exposure alone does not discriminate between the nonshivering capacity in mice and their ability to use muscle shivering to maintain their core temperature. To test nonshivering capacity of *LysM-Cre:Nrp1*^*fl/fl*^ mice, we treated them with the β3-adrenergic agonist CL316,243 or vehicle, and evaluated iBAT response (Fig. 4A, Supplemental Figure 3A). After treatment with CL316,243 both *LysM-Cre:Nrp1*^*fl/fl*^ and *LysM-Cre:Nrp1*^*wt/wt*^ mice had a significant decrease in body weight compared to vehicle-treated littermates (Fig. 4B, Supplemental Figure 3B). Despite CL316,243 inducing weight loss in both strains, the weight of *LysM-Cre:Nrp1*^*fl/fl*^ mice remained significantly higher when compared to control mice (Supplemental Figure 3C). Interestingly, treatment with CL316,243 was able to decrease the total and normalized weight of iBAT in *LysM-Cre:Nrp1*^*fl/fl*^ mice. iBAT weights in *LysM-Cre:Nrp1*^*fl/fl*^ mice became similar to the ones of *LysM-Cre:Nrp1*^*wt/wt*^ mice, whereas iWAT and eWAT were significantly increased (Fig. 4C–E, Supplemental Figure 3D–F). We verified this by means of H&E staining and showed that treatment with CL316,243 was able to reverse the hypertrophy observed in HFD-fed *LysM-Cre:Nrp1*^*fl/fl*^ mice (Fig. 4F). These data suggest that iBAT from *LysM-Cre:Nrp1*^*fl/fl*^ mice remains responsive to β3-adrenergic stimulation, suggesting it retained the ability to induce nonshivering thermogenesis.

## Discussion

In this study, we demonstrate that macrophages residing in interscapular brown adipose tissue express high levels of *Nrp1* compared to other tissue-resident macrophages. Given the metabolic role of BAT during obesity, we investigated the impact of macrophage-resident NRP1 deficiency on the thermogenic function of iBAT in a HFD model of weight gain. Our findings suggest that upon HFD, myeloid-resident NRP1 influences iBAT hypertrophy, impacting vasculature morphology, sympathetic innervation density and ultimately core temperature when exposed to a cold challenge. Mouse activity as evaluated by beam breaks as well as RER showed similar rates in both strains suggesting that the reduced ability of *LysM-Cre:Nrp1*^*fl/fl*^ mice to maintain body temperature in a cold challenge was not due to differences in mouse activity or substrate consumption. Although no differences were detected in energy expenditure between *LysM-Cre:Nrp1*^*fl/fl*^ and *LysM-Cre:Nrp1*^*wt/wt*^ mice, we observed that iBAT from *LysM-Cre:Nrp1*^*fl/fl*^ mice is responsive to β3-adrenergic stimulation and hence these mice are able to induce nonshivering thermogenesis.

Adipose tissue is subjected to tight immune regulation with studies having predominantly focused on immune regulation of WAT in obesity and diabetes. We previously showed that NRP1-expressing myeloid cells in WAT contribute to weight gain, insulin sensitivity and modulate metabolic homeostasis^22^. Furthermore, NRP1 deficiency in white adipose tissue macrophages as well as in peritoneal macrophages led to an increase in a polarization towards a classic pro-inflammatory phenotype^22^. With regards to iBAT, alternatively activated macrophages have been shown to play a role in the modulation of BAT thermogenesis through catecholamine synthesis^20^. However, these findings have been challenged with the finding that there are insufficient levels of tyrosine hydroxylase to synthesize relevant amounts of catecholamines in alternatively activated macrophages^21^, raising questions about the underlying signaling pathways in macrophages that could mediate BAT activation. Nonetheless, other studies showed that mutations associated with BAT resident macrophages influenced BAT innervation as well as thermogenesis^26,33,40^. The importance of innervation in iBAT has been demonstrated by denervation studies in animals subjected to cold exposure or HFD where it leads to a reduction in UCP1 expression levels, mitochondrial activity, blood flow and glucose uptake^41,42^. Moreover, in obesity, alterations in BAT vascular density can lead to hypoxia and mitochondrial dysfunction resulting in lipid droplet accumulation^43^. Given the critical role of NRP1 in myeloid-driven angiogenesis^23,44^, lipid uptake and mitochondrial lipid utilization^45^, it is possible that NRP1-expressing myeloid cells are influencing BAT homeostasis through vascular networks and local lipid metabolism. Future investigation on the discrepancies in the expression of thermogenic markers between *LysM-Cre:Nrp1*^*wt/wt*^ and *LysM-Cre:Nrp1*^*fl/fl*^ will provide insight on the role NRP1-expressing myeloid cells in iBAT function. Furthermore, while we focused on iBAT, iWAT is also reported to produce heat following browning of the tissue, hence future work could explore the contribution of myeloid cells expressing NRP1 to iWAT-mediated thermogenesis and browning.

In sum, our study suggests an indirect role for myeloid cells expressing NRP1 in BAT homeostasis during diet-induced obesity. Our data further supports the importance of vasculature^43^ and innervation^41,42^ in BAT homeostasis. Overall, we identify a distal influence of NRP1-expressing myeloid cells on iBAT and highlight the importance of immune regulation in brown adipose tissue.

## Methods

### Animal model

All experimental procedures adhered to the ARRIVE guidelines and were approved by the Animal Care Committee of the Maisonneuve-Rosemont Hospital Research Center and in accordance to the guidelines of the Canadian Council on Animal Care. Neuropilin-1 floxed mice (*B6.129(SJL)-Nrp1*^*tm2Ddg/*^*J*) were purchased from The Jackson Laboratory and crossed with *LysM-Cre:Nrp1*^*wt/wt*^ mice (*B6.129P2-Lyz2*/J) to generate myeloid-specific Nrp1-deficient mice (*LysM-Cre:Nrp1*^*fl/fl*^). Unless indicated otherwise, mice were bred and housed at 22ºC with ad libitum access to standard laboratory chow and water under a 12 h light/dark cycle.

### Study design

8-week-old *LysM-Cre:Nrp1*^*wt/wt*^ and *LysM-Cre:Nrp1*^*fl/fl*^ male mice were placed on either a regular-chow diet (RD) (10% kcal fat, 70% kcal carbohydrate, 20% kcal protein; Research diet D12450J) or a high-fat-diet (HFD) (60% kcal fat, 20% kcal carbohydrate, 20% kcal protein; Research diet D12492). During the experimental time-course of 12 weeks of diet, animals were weighted weekly. At 0 and 12 weeks of diet *LysM-Cre:Nrp1*^*wt/wt*^ and *LysM-Cre:Nrp1*^*fl/fl*^ mice were sedated with isoflurane gas and euthanized by cervical dislocation before proceeding to adipose tissue collection. Adipose depots were collected as follows: epididymal white adipose tissue (eWAT): bilateral intra-abdominal visceral depot attached to the epididymis; interscapular brown adipose tissue (iBAT), bilobed tissue between the scapulae;iInguinal white adipose tissue (iWAT), bilateral superficial subcutaneous between the skin and muscle fascia just anterior to the lower segment of the hind limbs. In most cases, an n = 5 was the minimum number of mice used. Age-matched littermates were randomly distributed in the different experimental groups. All experiments were reproduced in independent cohorts at least twice.

### Bioinformatics analysis with DESeq2, GSEA, GSVA and GO enrichment data analysis

Differential expression analysis was performed with DESeq2 and used for pre-ranked gene set enrichment analysis (GSEA 4.0.3). Genes that were exclusively upregulated or downregulated in a minimum of 6 out of 7 tissues were considered to determine the iBAT macrophages-specific signature. GSVA analysis was performed in R (v3.6.0.). Gene ontology (GO) analysis was performed with PANTHER classification system^49–51^ using the GO aspect: biological processes. Biological processes with a with FDR-adjusted *P* value (< 0.05) were considered as significant.

### 3D fluorescence-imaging

Collected adipose tissue was processed as previously reported^36^. Briefly, the tissues were fixed in 4% PFA overnight at RT followed by three washes with PBS of 1 h each. The tissues were dehydrated at RT in 20% methanol (in ddH2O) for 30 min, 40% methanol (in ddH2O) for 30 min, 60% methanol (in ddH2O) for 30 min, 80% methanol (in ddH2O) for 30 min and 100% methanol for 30 min twice. Then tissues were bleached with 5%H_2_O_2_ (1 volume of H_2_O_2_ diluted in 5 volumes of 100% methanol) containing EDTA-Na PH 8.0 at 4°C for 48 h. Tissues were rehydrated at RT in 80% methanol (in ddH2O) for 30 min, 60% methanol (in ddH2O) for 30 min, 40% methanol (in ddH2O) for 30 min, 20% methanol (in ddH2O) for 30 min and PBS/0.2% Triton X-100 for 1 h twice. The tissues were permeabilized with PBS/0.2% Triton X-100/20% DMSO/0.3 M glycine at 37°C for 24 h. Tissues were blocked with PBS/0.2% Triton X-100/10% DMSO/5% BSA at 37 °C for 24 h. Immunolabeling was performed with the primary antibodies (indicated in Supplemental Table S6) diluted in PBS/0.2% Tween-20/10 µg/mL heparin/5% DMSO/5% BSA at 37 °C for 72 h. Tissues were washed five times for 1 h with PBS/0.2% Tween-20/10ug/mL heparin at 37°C. Tissues were immunolabeled with corresponding Alexa Fluor dye-conjugated secondary antibodies (indicated in Supplemental Table S6) diluted in PBS/0.2% Tween-20/10 µg/mL heparin/5% DMSO/5% BSA at 37°C for 1 week. Tissues were washed five times for 2 h with PBS/0.2% Tween-20/10 µg/mL heparin at 37°C, followed by a O/N washing at 4°C. Immunolabeled adipose tissues were embedded in 1% agarose-blocks prepared in PBS followed by dehydration at RT in 20%, 40%, 60%, 80% methanol (diluted in ddH2O) for 1 h and 100% methanol for 1 h twice. Tissue blocks were incubated in a mixture of dichloromethane/methanol (2 volumes/1 volume) for 3 h, followed by dichloromethane for 15 min twice and cleared with 100% dibenzyl-ether for 1 h twice.

### 3D imaging acquisition and quantification

The optically cleared adipose tissues were imaged on a LaVision Light Sheet Ultramicroscope equipped with an Andor Neo sCMOS camera and a MVPLaPO 2x/0.50 objective lens and a 6 mm with medium length dipping cap and a 4 mm with long dipping cap. Version v328 of Imspector Microscope controller software was used. The whole view of the samples was imaged at a 0.63X effective magnification (1.26 × zoom) and scanned by two lightsheets by the left or right side with a step size of 3 µm. For imaging at 4X effective magnification (8 × zoom) and scanned by two lightsheets by the left or right side with a step size of 2 µm. The image stacks were acquired by continuous lighsheet scanning method without the contrast-blending algorithm.

Imaris v64 (9.1.2), Imaris software available at: http://www.bitplane.com/imaris/imaris was used to reconstruct the image stacks obtained from the volume imaging. The representative images of iBAT were acquired with the orthogonal perspective of the image stacks. Sympathetic fiber length and overall fiber density by total iBAT weight were obtained by multiplying the respective values by total iBAT wet weight.

### Histology analysis

Collected adipose tissue was fixed in Formalin 10% followed by standardized paraffin-embedding. Paraffin-embedded tissues were cut into 12 µm thick sections. Samples were deparaffinized and rehydrated and stained with Harris hematoxylin and eosin (H&E), followed by dehydration and mounting with PERTEX (HistoLab Products AB). For each sample, representative DIC images were taken with a Zeiss Axio-Imager Z2 (Zeiss) with a coupled AxioCam ICc 1 (Zeiss). Zeiss Axio Vision software (Zeiss) was used for image processing and editing.

For immunofluorescence, tissues were fixed in 4% paraformaldehyde overnight at 4°C, incubated with 30% PBS-sucrose for at least 48 h at 4°C and flash-frozen in OCT with liquid nitrogen before cryostat sectioning.

OCT-embedded tissues were cut into 12 µm thick sections. iBAT sections were permeabilized in methanol and blocked in 3% BSA, containing 0.05% Tween, 0.2% Triton for 1 h at room temperature in a humidified chamber. Followed by an overnight incubation with anti-rat CD31 [#550274, BD Biosciences] primary antibody (diluted at 1:500) in 3% BSA, containing 0.05% Tween, 0.2% Triton at 4 °C. Samples were incubated with Alexa Fluor secondary antibody at RT for 2 h. For each sample, Z-stacks were taken at 10 X on Olympus FV1000 confocal microscope.

### Vessel quantification

AngioTool^50^ analysis was performed on Z-stacks compressed on single images taken at 10X on an Olympus FV1000 confocal microscope. CD31 staining was measured.

Vessel area, total vessel length and vessel lacunarity by total iBAT weight were calculated by multiplying the respective values per total iBAT wet weight.

### Metabolic studies and comprehensive lab animal monitoring system (CLAMS)

Mice were implanted intraperitoneally with sterile temperature probes (G2 HR E-mitter, Bio-Lynx) 1 week prior to measurement of core temperature. Mice were placed in the CLAMS animal monitoring system for 24 h of acclimatization period, followed by 96 h of data collection at different temperatures. After the acclimatization, mice were exposed as follows: 24 h at room temperature, 48 h at 4°C and 24 h of recovery at room temperature. Indirect calorimetry, O_2_ consumption, CO_2_ production, RER, energy expenditure, food intake, water intake, and locomotor activity were measured during 96 h. Average food intake was determined by measuring the mean of food consumption (light and dark cycle) of *LysM-Cre:Nrp1*^*fl/fl*^ or *LysM-Cre:Nrp1*^*wt/wt*^ of mice at 10, 11 and 12 weeks of diet.

### Body composition

Lean and fat mass was examined by Echo MRI (Echo Medical Systems).

### β3-Adrenergic activation

Mice were injected intraperitoneally once daily for 4 consecutive days with CL316,243 at 1 mg/kg of weight. Saline was used as vehicle.

### Statistical methods

All results are presented as mean ± SEM. Analysis and statistical significance was analyzed using GraphPad Prism 5.0 (GraphPad Software; www.graphpad.com) by two-way ANOVA, when comparing multiple groups, and two-tailed unpaired Student’s t-test, when comparing only two groups. Statistical significance was considered when *p* < 0.05 and it is indicated as: *p < 0.05; **p < 0.01; ***p < 0.001. Biological experiment numbers were listed in figure legends.

## Supplementary Information

Supplementary Information 1.

Supplementary Information 2.

Supplementary Information 3.

Supplementary Information 4.

Supplementary Information 5.

Supplementary Information 6.

Supplementary Information 7.

Supplementary Information 8.

Supplementary Information 9.

Supplementary Information 10.

## Data Availability

Data, material and reagent information regarding this work can be inquired upon reasonable request to the corresponding author.

## References

[1] GBD Obesity Collaborators (2017). Health effects of overweight and obesity in 195 countries over 25 years. N. Engl. J. Med..

[2] NCD Risk Factor Collaboration (2016). Trends in adult body-mass index in 200 countries from 1975 to 2014: a pooled analysis of 1698 population-based measurement studies with 19.2 million participants. Lancet.

[3] Lau DC, Dhillon B, Yan H, Szmitko PE, Verma S (2005). Adipokines: molecular links between obesity and atheroslcerosis. Am. J. Physiol. Heart Circ. Physiol..

[4] Smith RE (1964). Thermoregulatory and adaptive behavior of brown adipose tissue. Science.

[5] Wu J (2012). Beige adipocytes are a distinct type of thermogenic fat cell in mouse and human. Cell.

[6] Park A, Kim WK, Bae KH (2014). Distinction of white, beige and brown adipocytes derived from mesenchymal stem cells. World J. Stem Cells.

[7] Cummins TD (2014). Metabolic remodeling of white adipose tissue in obesity. Am. J. Physiol. Endocrinol. Metab..

[8] Ravussin E, Galgani JE (2011). The implication of brown adipose tissue for humans. Annu. Rev. Nutr..

[9] Cypess AM (2015). Activation of human brown adipose tissue by a beta3-adrenergic receptor agonist. Cell Metab..

[10] Bartelt A (2011). Brown adipose tissue activity controls triglyceride clearance. Nat. Med..

[11] Mathis D (2013). Immunological goings-on in visceral adipose tissue. Cell Metab..

[12] Chung KJ, Nati M, Chavakis T, Chatzigeorgiou A (2018). Innate immune cells in the adipose tissue. Rev. Endocr. Metab. Disord..

[13] Kaminski DA, Randall TD (2010). Adaptive immunity and adipose tissue biology. Trends Immunol..

[14] Exley MA, Hand L, O'Shea D, Lynch L (2014). Interplay between the immune system and adipose tissue in obesity. J. Endocrinol..

[15] Kane H, Lynch L (2019). Innate immune control of adipose tissue homeostasis. Trends Immunol..

[16] Fitzgibbons TP, Czech MP (2016). Emerging evidence for beneficial macrophage functions in atherosclerosis and obesity-induced insulin resistance. J. Mol. Med. (Berl.).

[17] Mraz M, Haluzik M (2014). The role of adipose tissue immune cells in obesity and low-grade inflammation. J. Endocrinol..

[18] Weisberg SP (2003). Obesity is associated with macrophage accumulation in adipose tissue. J. Clin. Investig..

[19] Catrysse L, van Loo G (2018). Adipose tissue macrophages and their polarization in health and obesity. Cell Immunol..

[20] Nguyen KD (2011). Alternatively activated macrophages produce catecholamines to sustain adaptive thermogenesis. Nature.

[21] Fischer K (2017). Alternatively activated macrophages do not synthesize catecholamines or contribute to adipose tissue adaptive thermogenesis. Nat. Med..

[22] Wilson AM (2018). Neuropilin-1 expression in adipose tissue macrophages protects against obesity and metabolic syndrome. Sci. Immunol.

[23] Geretti E, Shimizu A, Klagsbrun M (2008). Neuropilin structure governs VEGF and semaphorin binding and regulates angiogenesis. Angiogenesis.

[24] Erskine L (2017). VEGF-A and neuropilin 1 (NRP1) shape axon projections in the developing CNS via dual roles in neurons and blood vessels. Development.

[25] Fantin A, Maden CH, Ruhrberg C (2009). Neuropilin ligands in vascular and neuronal patterning. Biochem. Soc. Trans..

[26] Wolf Y (2017). Brown-adipose-tissue macrophages control tissue innervation and homeostatic energy expenditure. Nat. Immunol..

[27] Yoneshiro T (2011). Age-related decrease in cold-activated brown adipose tissue and accumulation of body fat in healthy humans. Obesity (Silver Spring).

[28] Koksharova E (2017). The relationship between brown adipose tissue content in supraclavicular fat depots and insulin sensitivity in patients with type 2 diabetes mellitus and prediabetes. Diabetes Technol. Ther..

[29] Betz MJ, Enerback S (2015). Human brown adipose tissue: what we have learned so far. Diabetes.

[30] Cao Y (2007). Angiogenesis modulates adipogenesis and obesity. J. Clin. Investig..

[31] Sharon L., J., Yihai C. in *Angiogenesis in Adipose Tissue.* 77–102 (Springer, 2013).

[32] Cao Y, Wang H, Wang Q, Han X, Zeng W (2018). Three-dimensional volume fluorescence-imaging of vascular plasticity in adipose tissues. Mol. Metab..

[33] Rached MT (2019). Deletion of myeloid IRS2 enhances adipose tissue sympathetic nerve function and limits obesity. Mol. Metab..

[34] Bartness TJ, Vaughan CH, Song CK (2010). Sympathetic and sensory innervation of brown adipose tissue. Int. J. Obes. (Lond.).

[35] Francois M (2019). Sympathetic innervation of the interscapular brown adipose tissue in mouse. Ann. N. Y. Acad. Sci..

[36] Jiang H, Ding X, Cao Y, Wang H, Zeng W (2017). Dense intra-adipose sympathetic arborizations are essential for cold-induced beiging of mouse white adipose tissue. Cell Metab..

[37] Ramos-Jimenez A (2008). The respiratory exchange ratio is associated with fitness indicators both in trained and untrained men: a possible application for people with reduced exercise tolerance. Clin. Med. Circ. Respirat. Pulm. Med..

[38] Tschop MH (2011). A guide to analysis of mouse energy metabolism. Nat. Methods.

[39] Cannon B, Nedergaard J (2011). Nonshivering thermogenesis and its adequate measurement in metabolic studies. J. Exp. Biol..

[40] Pirzgalska RM (2017). Sympathetic neuron-associated macrophages contribute to obesity by importing and metabolizing norepinephrine. Nat. Med..

[41] Fischer AW, Schlein C, Cannon B, Heeren J, Nedergaard J (2019). Intact innervation is essential for diet-induced recruitment of brown adipose tissue. Am. J. Physiol. Endocrinol. Metab..

[42] Wang W, Seale P (2016). Control of brown and beige fat development. Nat. Rev. Mol. Cell Biol..

[43] Shimizu I (2014). Vascular rarefaction mediates whitening of brown fat in obesity. J. Clin. Investig..

[44] Staton CA, Kumar I, Reed MW, Brown NJ (2007). Neuropilins in physiological and pathological angiogenesis. J. Pathol..

[45] Kawasaki T (2002). Requirement of neuropilin 1-mediated Sema3A signals in patterning of the sympathetic nervous system. Development.

[46] Dejda A (2014). Neuropilin-1 mediates myeloid cell chemoattraction and influences retinal neuroimmune crosstalk. J. Clin. Investig..

[47] Casazza A (2013). Impeding macrophage entry into hypoxic tumor areas by Sema3A/Nrp1 signaling blockade inhibits angiogenesis and restores antitumor immunity. Cancer Cell.

[48] Bostrom P (2012). A PGC1-alpha-dependent myokine that drives brown-fat-like development of white fat and thermogenesis. Nature.

[49] Ashburner M (2000). Gene ontology: tool for the unification of biology. The Gene Ontology Consortium. Nat. Genet..

[50] The Gene Ontology, C. The Gene Ontology Resource: 20 years and still GOing strong. *Nucleic Acids Res.* **47**, D330–D338. 10.1093/nar/gky1055 (2019). 10.1093/nar/gky1055 PMC6323945 30395331

[51] Mi H, Muruganujan A, Ebert D, Huang X, Thomas PD (2019). PANTHER version 14: more genomes, a new PANTHER GO-slim and improvements in enrichment analysis tools. Nucleic Acids Res..

[52] Zudaire E, Gambardella L, Kurcz C, Vermeren S (2011). A computational tool for quantitative analysis of vascular networks. PLoS ONE.

