# Supplementary material for: Myeloid-resident neuropilin-1 influences brown adipose tissue in obesity
Source: Sci Rep. 2021 Aug 3;11:15767. doi: 10.1038/s41598-021-95064-w (PMC8333363; doi:10.1038/s41598-021-95064-w)
Supplement: Supplementary file 1 — Supplementary Information 1. [file 41598_2021_95064_MOESM1_ESM.pdf]

SUPPLEMENTARY FILES

Title: Myeloid-resident neuropilin-1 influences brown adipose tissue in obesity

Roberto Diaz Marin[1], Sergio Crespo-Garcia[1], Ariel M Wilson[2], Manuel Buscarlet[1], Agnieszka Dejda[2], Frédérik Fournier[1], Rachel Juneau[1], Thierry Alquier[3], Przemyslaw Sapieha[1,2 *]

[1]Department of Biochemistry, Maisonneuve-Rosemont Hospital Research Centre, Université de Montréal, Montréal, Québec H1T2M4, Canada

[2]Department of Ophthalmology, Maisonneuve-Rosemont Research Centre, Université de Montréal, Montréal, Québec H1T2M4, Canada

[3]Montreal Diabetes Research Centre and Centre de Recherche du Centre Hospitalier de l'Université de Montréal (CRCHUM), 900 rue Saint-Denis, Montréal, QC, H2X0A9, Canada.

*Corresponding author

Running title: Myeloid-resident NRP1 influences BAT in obesity

Supplemental Figures Legends

Supplemental Figure S1, related to Figure2. Myeloid-resident NRP1 influences iBAT mass and whole body weight. A. Total daily food intake and **B**. Total daily energy intake of *LysM-Cre:Nrp1*$^{wt/wt}$ and *LysM-Cre:Nrp1*$^{fl/fl}$ mice fed a high fat diet after 11 weeks of diet, n=4-7 mice per genotype. **C.** Schematic representation of the collected adipose tissues in mouse: epididymal WAT, interscapular BAT and inguinal WAT. **D.** iBAT, **E**. iWAT and **F**. eWAT masses were calculated as a ratio from tissue-specific weight over total bodyweight at different time-points after RD or HFD diet feeding (n=4-6). **G.** Comparison of the body weight of *LysM-Cre:Nrp1*$^{wt/wt}$ and *LysM-Cre:Nrp1*$^{fl/fl}$ mice at the beginning of the diet (still in Normal chow (NC)) and after 12 weeks of HFD. **H.** iBAT, **I**. iWAT and **J**. eWAT masses were calculated as a ratio from tissue-specific weight over total bodyweight at different time-points after 4 weeks of RD or HFD diet feeding (n=6-8). **K.** iBAT, **L**. iWAT and **M**. eWAT masses; *LysM-Cre:Nrp1*$^{wt/wt}$ (n=5) and *LysM-Cre:Nrp1*$^{fl/fl}$ (n=5) after 4 weeks of diet. **N-O.** Quantification of vessel area (**N**) and length (**O**) by total iBAT weight normalized to *LysM-Cre:Nrp1*$^{wt/wt}$ (n=5 per group). **P.** Quantification of sympathetic fiber length by total iBAT weight from 12-week HFD-fed *LysM-Cre:Nrp1*$^{wt/wt}$ and *LysM-Cre:Nrp1*$^{fl/fl}$ mice housed at RT. Data are presented as mean ± SEM. * p < 0.05, ** p < 0.01 and *** p < 0.001.

Supplemental Figure S2, related to Figure 3. Deficiency in Myeloid-resident NRP1 does not influence iBAT energy expenditure. Light and dark energy expenditure (area under the curve) during **A.** room temperature (22°C), **B.** cold (4°C), **C.** return to room temperature (22°C) exposure of *LysM-Cre:Nrp1*$^{wt/wt}$ and *LysM-Cre:Nrp1*$^{fl/fl}$ mice 12 weeks of HFD, n=4-7 mice per genotype. **D.** Echo MRI, **E.** Fat mass percentage of *LysM-Cre:Nrp1*$^{wt/wt}$ and *LysM-Cre:Nrp1*$^{fl/fl}$ mice fed a high fat diet after 11 weeks of diet, n=4-7 mice per genotype, . Data are presented as mean ± SEM. * p < 0.05 and ** p < 0.01.

Supplemental Figure S3, related to Figure 4. Myeloid-resident NRP1 influences iBAT sympathetic tone.

A. Weight gain of *LysM-Cre:Nrp1*$^{fl/fl}$, *LysM-Cre:Nrp1*$^{wt/wt}$ mice on RD or HFD for 12 weeks (n=10-12). **B.** Weight difference of *LysM-Cre:Nrp1*$^{fl/fl}$ and *LysM-Cre:Nrp1*$^{wt/wt}$ mice treated with CL316,243 or vehicle after 4 days (n=3-4). **C.** Comparison of the body weight of *LysM-Cre:Nrp1*$^{wt/wt}$ and *LysM-Cre:Nrp1*$^{fl/fl}$ mice treated with CL316,243 or vehicle (n=5-7). **D.** iBAT, **E.** iWAT and **F.** eWAT masses were calculated as a ratio from tissue-specific weight over total bodyweight at 12 weeks of HFD following 4 days treatment with CL316,243 or vehicle (n=4-6). Data are presented as mean ± SEM. * $p < 0.05$, ** $p < 0.01$ and *** $p < 0.001$.

__Supplemental Tables__

Supplemental Table S1. GSEA analysis of iBAT macrophages across macrophages populations

Supplemental Table S2. List of genes significantly upregulated in iBAT macrophages

Supplemental Table S3. List of genes significantly downregulated in iBAT macrophages

Supplemental Table S4. Top 15 biological processes with upregulated genes in iBAT macrophages specific signature

Supplemental Table S5. Gene frequency of genes associated with the top 15 of biological processes from upregulated genes in iBAT macrophages specific signature

Supplemental Table S6. Antibodies used for immunofluorescence
