# Supplementary figures and images for: Myeloid-resident neuropilin-1 influences brown adipose tissue in obesity
Source: Sci Rep. 2021 Aug 3;11:15767. doi: 10.1038/s41598-021-95064-w (PMC8333363; doi:10.1038/s41598-021-95064-w)

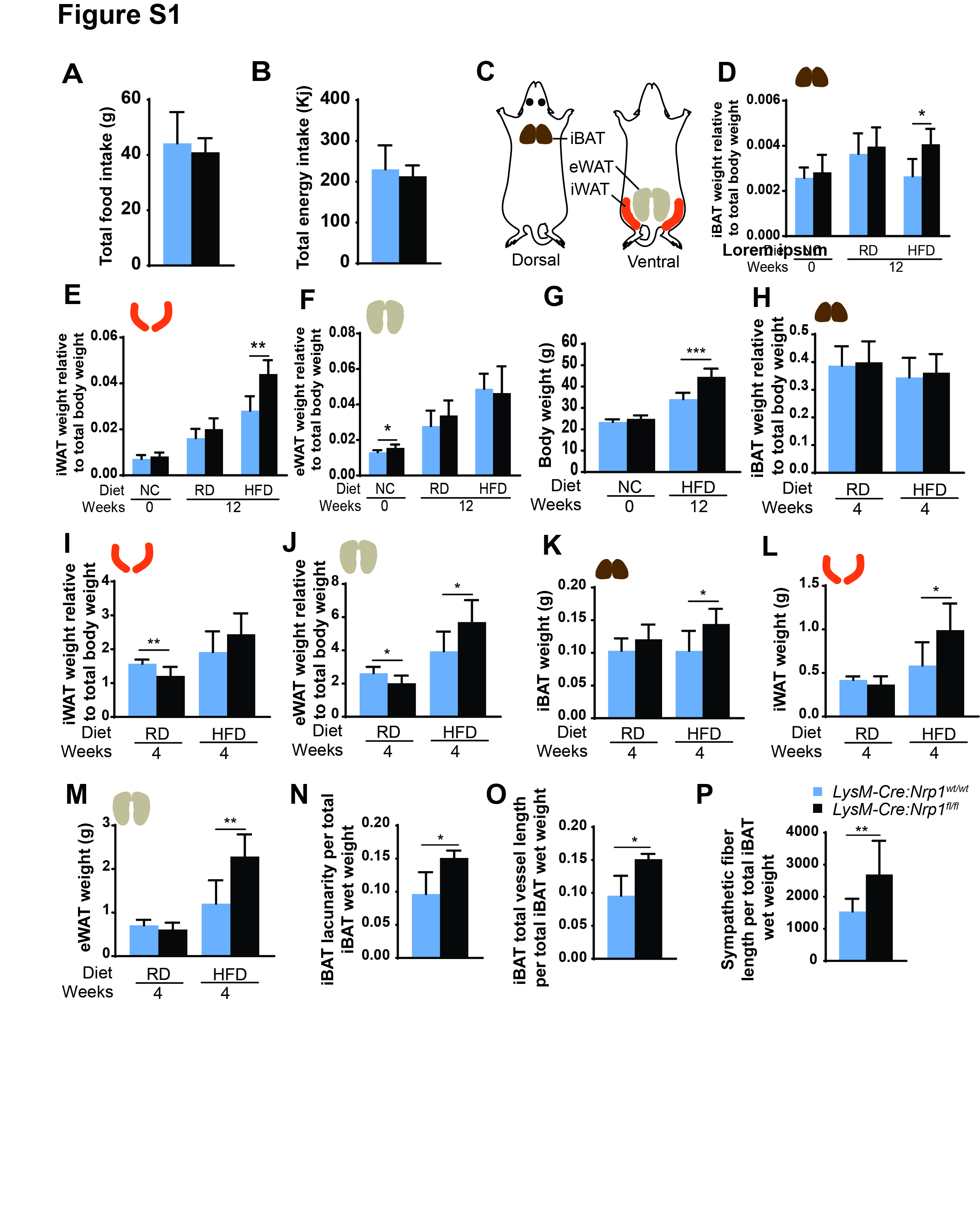

Supplement: Supplementary file 2 — Supplementary Information 2. [file 41598_2021_95064_MOESM2_ESM.jpg]

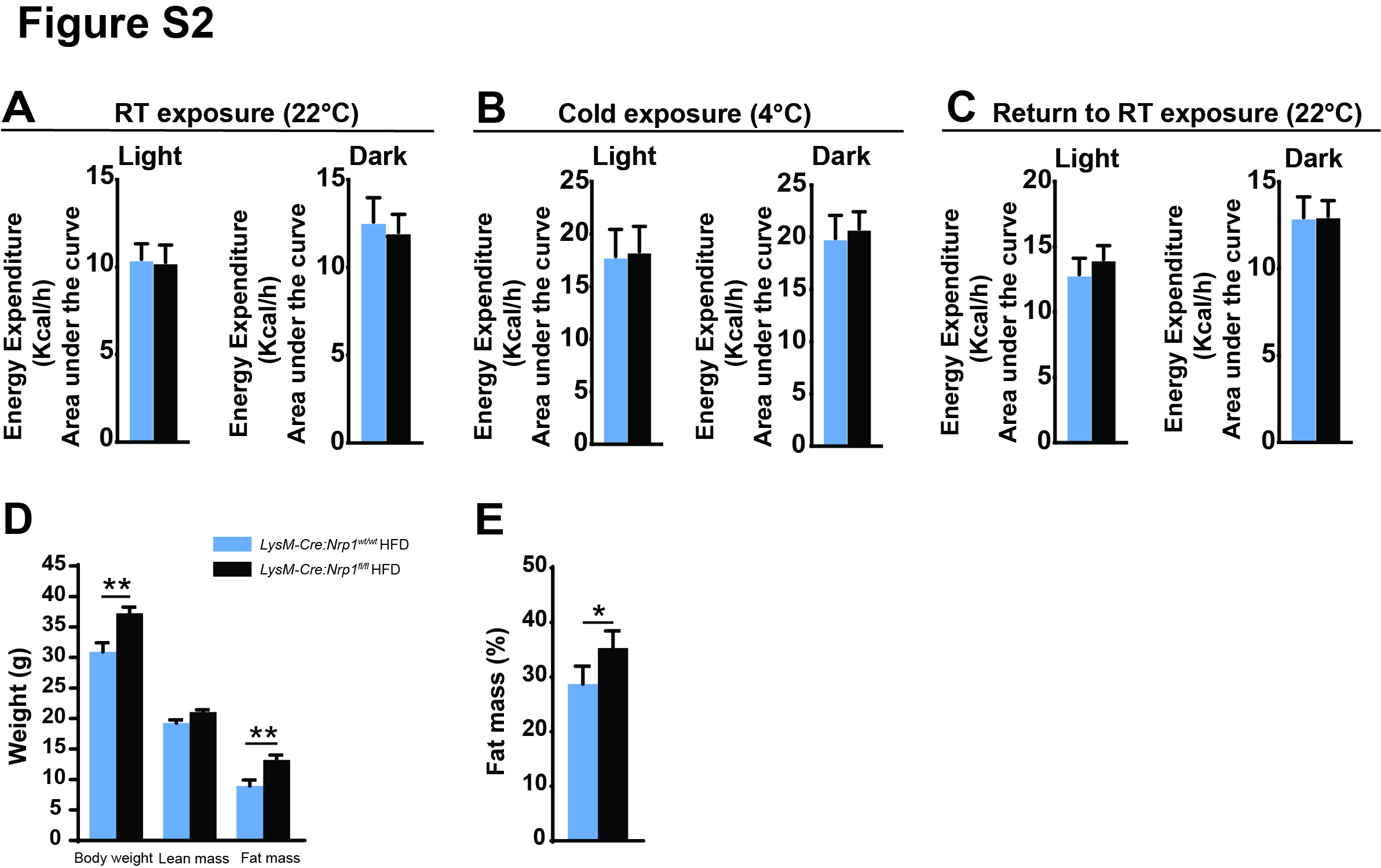

Supplement: Supplementary file 3 — Supplementary Information 3. [file 41598_2021_95064_MOESM3_ESM.jpg]

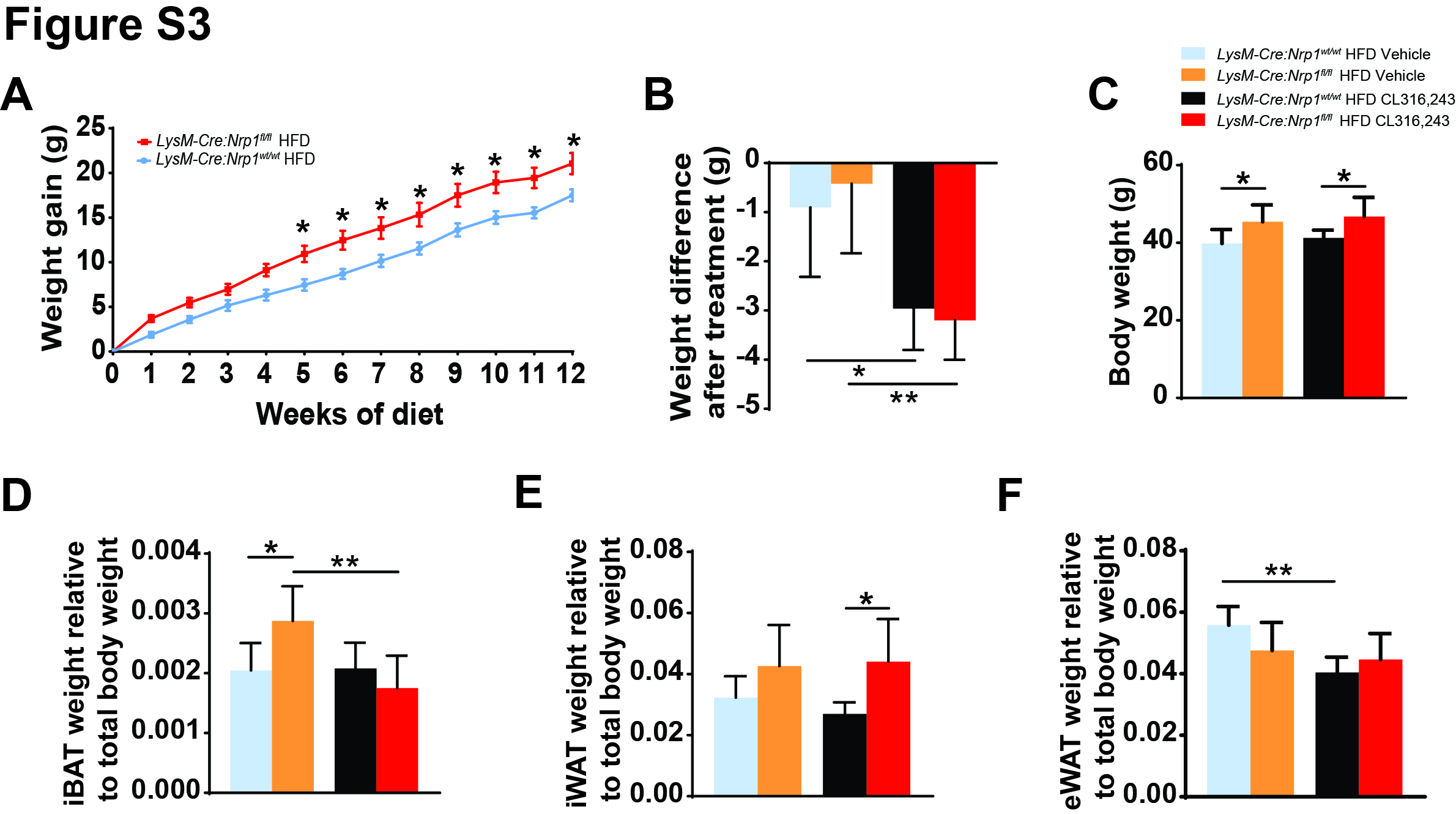

Supplement: Supplementary file 4 — Supplementary Information 4. [file 41598_2021_95064_MOESM4_ESM.jpg]
